# Supplementary material for: Degree of Hydrolysis Regulated by Enzyme Mediation of Wheat Gluten Fibrillation: Structural Characterization and Analysis of the Mechanism of Action
Source: Int J Mol Sci. 2023 Aug 31;24(17):13529. doi: 10.3390/ijms241713529 (PMC10488075; doi:10.3390/ijms241713529)
Supplement: Supplementary file 1 [file ijms-24-13529-s001.zip › ijms-2566235-supplementary.pdf]

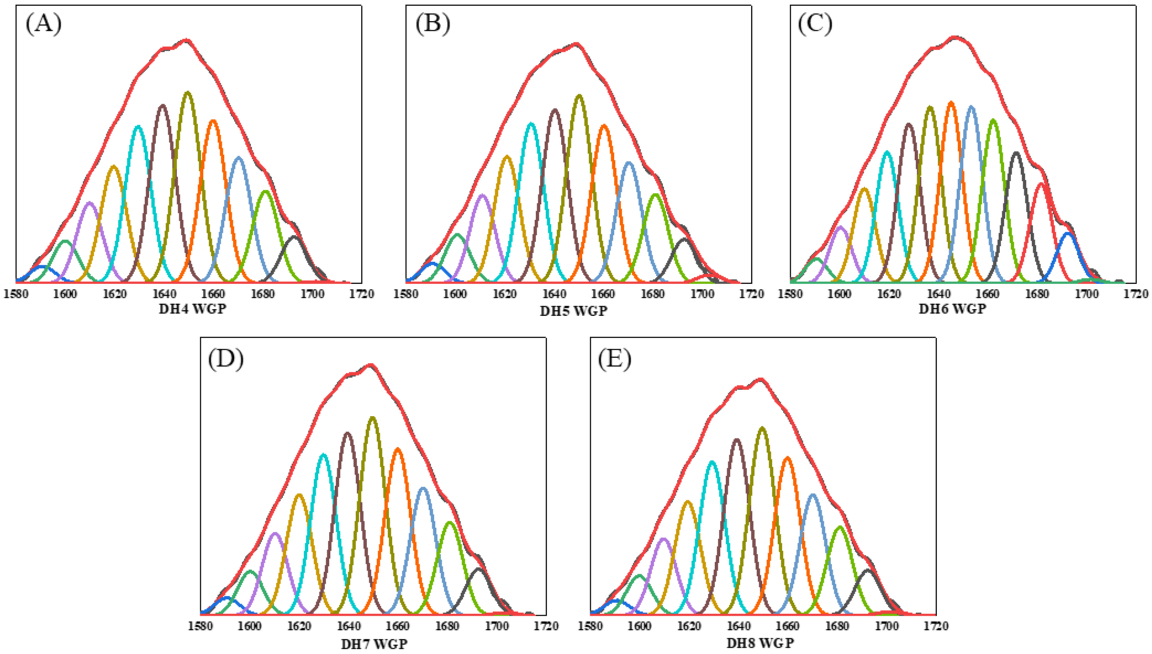

Figure S1. Fitting diagram of the amide I region for DH4-8 WGP.

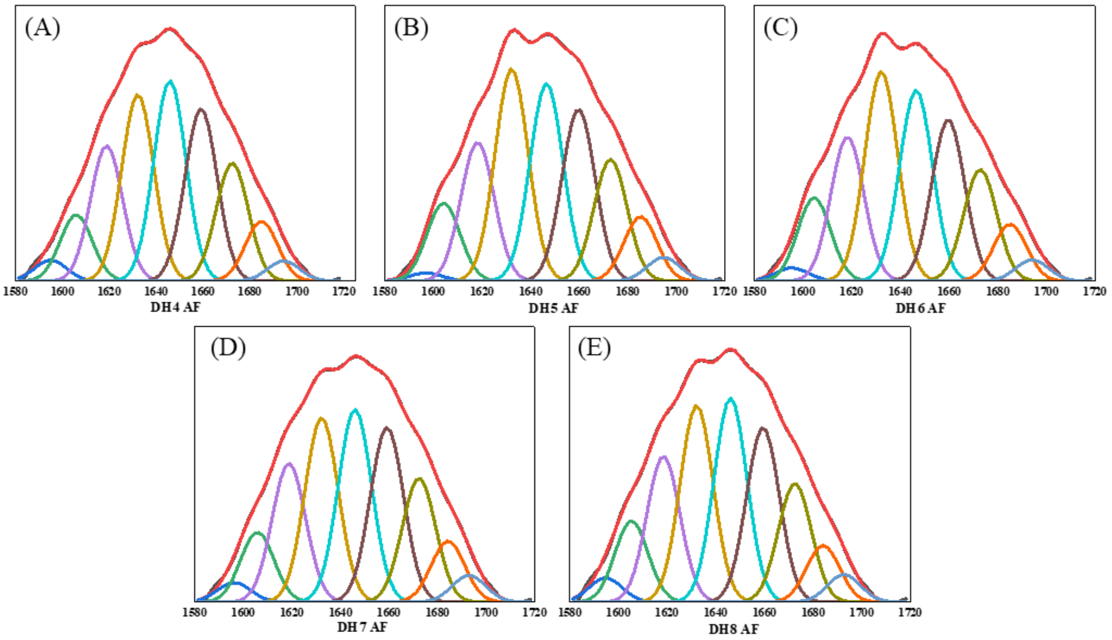

Figure S2. Fitting diagram of the amide I region for DH4-8 AF.
